# Intramyocardial Delivery of Mesenchymal Stem Cell-Seeded Hydrogel Preserves Cardiac Function and Attenuates Ventricular Remodeling after Myocardial Infarction

**DOI:** 10.1371/journal.pone.0051991

**Published:** 2012-12-20

**Authors:** Eva Mathieu, Guillaume Lamirault, Claire Toquet, Pierre Lhommet, Emilie Rederstorff, Sophie Sourice, Kevin Biteau, Philippe Hulin, Virginie Forest, Pierre Weiss, Jérôme Guicheux, Patricia Lemarchand

**Affiliations:** 1 INSERM UMR1087, CNRS UMR6291, l’institut du thorax, Nantes, France; 2 Université de Nantes, Structure Fédérative de Recherche Santé F. Bonamy, Nantes, France; 3 CHU de Nantes, Nantes, France; 4 Service d’Anatomie Pathologique, E.A. Biometadys, CHU de Nantes, Nantes, France; 5 INSERM, U791, Laboratory of Osteo-Articular and Dental Tissue Engineering, Group STEP “Skeletal tissue Engineering and Physiopathology”, Nantes, France; 6 Cellular and Tissular Imaging Core Facility of Nantes University (MicroPICell), Nantes, France; Institute of Clinical Medicine, National Cheng Kung University, Taiwan

## Abstract

**Background:**

To improve the efficacy of bone marrow-derived mesenchymal stem cell (MSC) therapy targeted to infarcted myocardium, we investigated whether a self-setting silanized hydroxypropyl methylcellulose (Si-HPMC) hydrogel seeded with MSC (MSC+hydrogel) could preserve cardiac function and attenuate left ventricular (LV) remodeling during an 8-week follow-up study in a rat model of myocardial infarction (MI).

**Methodology/Principal Finding:**

Si-HPMC hydrogel alone, MSC alone or MSC+hydrogel were injected into the myocardium immediately after coronary artery ligation in female Lewis rats. Animals in the MSC+hydrogel group showed an increase in cardiac function up to 28 days after MI and a mid-term prevention of cardiac function alteration at day 56. Histological analyses indicated that the injection of MSC+hydrogel induced a decrease in MI size and an increase in scar thickness and ultimately limited the transmural extent of MI. These findings show that intramyocardial injection of MSC+hydrogel induced short-term recovery of ventricular function and mid-term attenuation of remodeling after MI.

**Conclusion/Significance:**

These beneficial effects may be related to the specific scaffolding properties of the Si-HPMC hydrogel that may provide the ability to support MSC injection and engraftment within myocardium.

## Introduction

After myocardial infarction (MI), left ventricular (LV) remodeling occurs with early and progressive extracellular matrix (ECM) degradation, infarct zone expansion, scar thinning, LV enlargement and eventually, transition to heart failure [1], [2]. Current anti-remodeling therapies are limited as they fail to prevent ventricle enlargement [3], [4] and morbidity-mortality remains high [5]. Mesenchymal stem cell (MSC) injection into the infarcted myocardium is a newly developed strategy for cardiac tissue repair and regeneration after MI [6]. The beneficial effects of MSC injection have been partly related to their paracrine activity. MSC secrete angiogenic, anti-apoptotic, and anti-inflammatory cytokines that may contribute to the recovery of cardiac function [7], [8] and significantly decrease fibrosis of the myocardium [9], [10]. Most MSC administration strategies use intramyocardial injections of cells suspended in culture medium. However, this technique is limited by low cell retention and survival rates. For example, several studies have shown that more than 80%–90% of grafted cells die within 72 hours after injection into the myocardium [11], [12]. Multiple mechanisms may contribute to the premature death of grafted cells, including oxidative stress, hypoxia, and inflammation [13], [14]. Recently, cardiac tissue engineering, combining cells and scaffolding biomaterials, has emerged as a promising approach to provide support for tissue repair after MI [15], [16]. Among the various types of biomaterials currently available, hydrogels comprising hydrophilic, biocompatible polymers and peptides may represent excellent cell delivery systems due to their unique property of permitting *in situ* gel formation [17]. There are two major types of hydrogels: natural hydrogels, such as fibrin glue [18] and alginate [19], and synthetic hydrogels, such as polyethylene glycol (PEG) [20]. Natural hydrogels are used as scaffolds because they exhibit several critical biological functions that synthetic polymers lack, such as cell adhesion and biodegradation. We previously designed a water-rich hydrogel consisting of silanized hydroxypropyl methylcellulose (Si-HPMC) that can be steam sterilized [21] and supports the diffusion of signaling molecules and nutrients [22]. Interestingly, this Si-HPMC hydrogel can be injected together with MSC *in vivo* and is able to self-crosslink to form a scaffolding matrix [23].

In this context, and to develop a novel cardiac tissue engineering strategy, we questioned whether a hydrogel-assisted intramyocardial injection of bone marrow-derived MSC could attenuate post-MI cardiac disorders. To address this issue, we used a rat model to evaluate the effects of injecting Si-HPMC hydrogel seeded with MSC on cardiac function and LV remodeling in a rat model of MI.

## Results

### Rheological Characteristics of Si-HPMC Hydrogel

Rheological properties of Si-HPMC solution mixed with acidic buffer (1∶1) were measured. The final product (Si-HPMC hydrogel) was a reticulated hydrogel with a pH value of 7.4 after 27.2±3.4 min. Dynamic rheological measurements were performed to characterize the Si-HPMC hydrogel, including shear strain measurements to evaluate the storage modulus (G’, which characterizes the hard component), and the loss modulus (G”, which characterizes the liquid component). After three weeks of cross-linking and completion of the self-setting process, the G’ value was 343.2±106.5 Pa and the G” was 44.5±15.4 Pa. The compressive modulus (which reflects the stiffness of the material in a compressive experiment) at a 5% strain was 328.6±97.0 Pa.

### MSC Characterization and Viability in Three-dimensional Culture within the Si-HPMC Hydrogel

**Figure 1:**
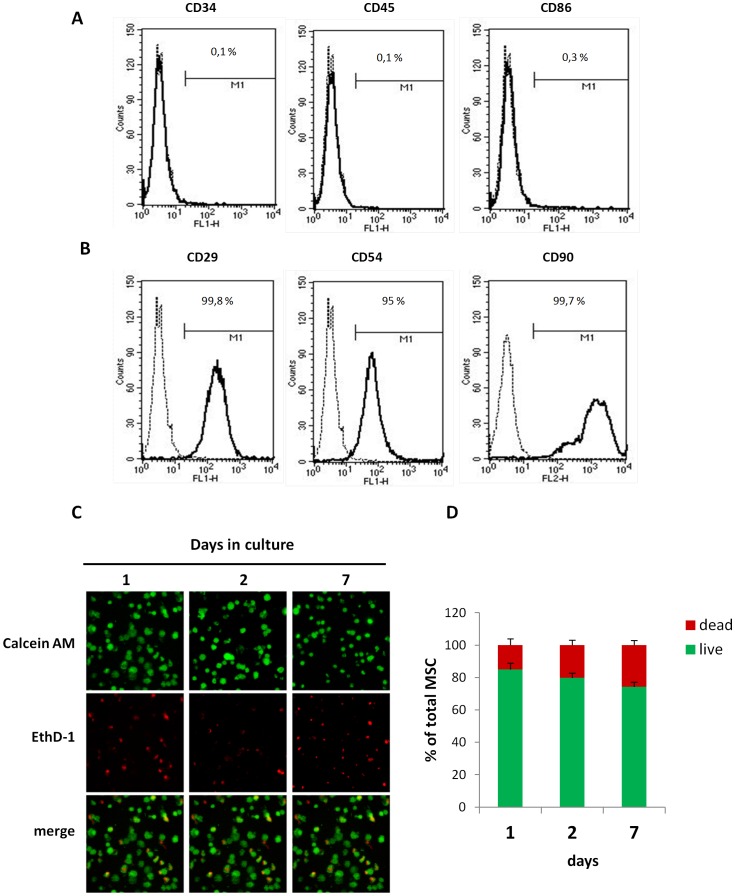
MSC characterization and viability in 3D culture within the Si-HPMC hydrogel. (A and B) Flow cytometric analysis of MSC for CD29, CD54, CD90, CD34, CD45 and CD86 expression. 10,000 events were scored. Results are expressed as % of positive cells in the whole population on representative histogram plots. (C and D) MSC were cultured in 3D Si-HPMC hydrogel for the indicated times. (C) Labeling cells with calcein-AM (green color) and with EthD-1 (red color) revealed living and dead cells, respectively. Representative samples of MSC cultures visualized by confocal microscopy. (D) As described in the Materials section, the percentages of living and dead MSC cultured in 3D within hydrogel over 7 days (*p* = NS as compared between time points, one-way ANOVA). All values represent mean ± SEM. Scale bar = 100 µm.

To characterize MSCs before *in vivo* injection, the expression of a number of surface markers [24], [25] was assessed by flow cytometry. No expression of CD34 and CD45 was observed (Figure 1A). In contrast, MSC expressed CD29, CD54 and CD90 (Figure 1B).

To evaluate whether the Si-HPMC hydrogel was cytotoxic, MSC viability was monitored in Si-HPMC 3D-culture by confocal fluorescent microscopy (Figure 1C). MSC viability was assessed by measurement of green fluorescence intensity, as a consequence of incorporation of the calcein fluorescent probe into the cytoplasm (Figure 1D). The results showed that MSC viability was maintained throughout the experiment.

### MSC Tracking in Cardiac Tissue

**Figure 2:**
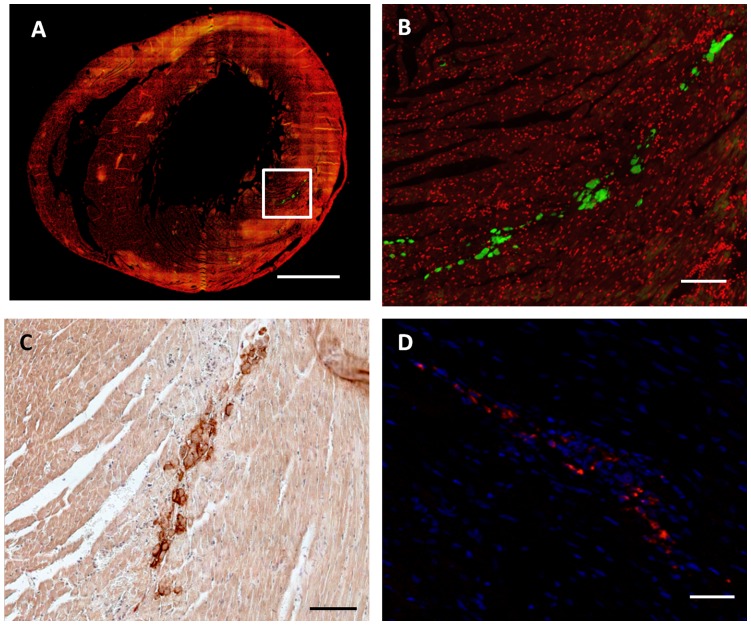
Evaluation of MSC engraftment 24 hours and 14 days after *in vivo* injection with Si-HPMC hydrogel into cardiac tissue. MSC engraftment 24 hours and 14 days after *in vivo* MSC+Si-HPMC hydrogel injection into cardiac tissue is shown on representative transversal histology heart sections. (A, B) Cell nuclei were labeled with To-Pro-3 (red fluorescence). MSC were labeled prior to injection with a fluorescent dye, CFSE (green fluorescence) and visualized 24 h after the implantation. (C) CD90 staining allowed identification of implanted MSC in left ventricle 24 h after injection. (D) PKH26 labeled MSC (red fluorescence) in heart wall with DAPI for cell nuclei (blue fluorescence), 14 days after implantation. (A) Scale bar = 1.5 mm. (B, C and D) scale bar Scale bar = 0.5 mm.

To determine whether the Si-HPMC hydrogel may allow cell injection and engraftment in cardiac tissue, MSC were detected 24 h after their implantation using CFSE fluorescence labeling (Figure 2A and 2B) and CD90 expression in host tissue (Figure 2C). Because of the possibility of a rapid loss of fluorescence of the CFSE- labeled MSCs, the red fluorescence of PKH26 was used to track MSC 14 days after the injection into the cardiac tissue (Figure 2D). Histological evaluation of the myocardium 24 h after MSC injection with or without hydrogel showed that most of the grafted MSC were localized in the left ventricular wall around injection sites and MSC survived 14 days after the injection into the left ventricle tissue. These data indicate that Si-HPMC hydrogel was able to support MSC implantation and engraftment in cardiac tissue.

### Comparative Effects of Hydrogel, MSC, and MSC+hydrogel on Cardiac Function and LV Remodeling

MI was induced by ligation of the LAD coronary artery in 62 rats. After MI induction, rats were randomized into 4 treatment groups to receive intramyocardial injections of (1) PBS as the control, (2) hydrogel, (3) MSC and (4) MSC+hydrogel. In our study, the overall mortality 24 hours after surgery was 30.7±7.7% (19/62 rats) with no significant differences between treatment groups (Table 1A). Echocardiography was performed 1 day after coronary artery ligation to exclude rats without a significant MI (defined as animals with a LVEF >70%; Table 1B). Importantly, the number of excluded rats was not significantly different between treatment groups (Table 1B), nor were the parameters of LV dimensions and function measured at Day 1 of the echocardiography follow-up analysis (Table 2).

**Table 1:** Number of animals included in the study.

	Animals number at baseline	Living animals at day 1
**PBS**	11	10
**hydrogel**	14	11
**MSC**	15	9
**MSC+hydrogel**	22	13
**Total**	62	43
	**Animals number with LVEF>70% at day 1**	**Animals number with LVEF≤70% at day 1**
**PBS**	4	6
**hydrogel**	4	7
**MSC**	1	8
**MSC+hydrogel**	4	9
**Total**	13	30

(A) Number of animals surviving 1 day post-MI. *p* = 0.21 (Fisher exact test). (B) Number of animals in the follow-up study with an EF≤70% 1 day after MI. *p* = 0.26 (Fisher exact test).

**Table 2:** Echocardiography measurements at baseline (Bsl) and at 1 day (d1), 7 days (d7), 28 days (d28) and 56 days (d56) after MI.

Parameters	PBS (n = 6)	hydrogel (n = 7)	MSCs (n = 8)	MSC+hydrogel (n = 9)
**LVEDD (mm)**				
Bsl	5.4±0.2	5.2±0.2	5.6±0.1	5.6±0.2
d1	5.9±0.1	5.8±0.3	6.2±0.2	6.0±0.1
d7	6.6±0.1	6.1±0.3	6.3±0.1	6.1±0.2
d28	7.2±0.2¥	6.9±0.3¥	7.0±0.3	6.6±0.3
d56	7.4±0.3¥	7.3±0.5¥	7.0±0.4	6.8±0.2
**LVESD (mm)**				
Bsl	2.4±0.1	2.6±0.2	2.8±0.1	3.0±0.2
d1	4.0±0.1	4.1±0.3	4.3±0.1	4.3±0.2
d7	5.0±0.1¥	4.0±0.3*	4.3±0.2*	3.9±0.2*
d28	5.7±0.3¥	5.0±0.3*	5.2±0.4*	4.3±0.3*
d56	6.0±0.3¥	5.5±0.5*	4.9±0.3*	4.8±0.1* $
**LVFS (%)**				
Bsl	56.6±1.7	49.4±2.0	49.5±1.0	47.1±2.2
d1	29.0±2.4	29.9±2.8	30.4±1.8	27.9±1.9
d7	24.1±0.9	34.1±2.0*	31.2±2.5	36.9±1.7¥ * +
d28	20.2±2.3¥	28.0±1.2*	26.7±3.3*	34.4±1.9¥ * ^+$^
d56	19.6±1.5¥	25.6±2.9*	30.8±2.4*	29.4±1.5* $
**LVEF (%)**				
Bsl	87.4±1.5	86.0±1.2	86.8±1.9	88.2±1.5
d1	61.3±4.0	64.6±2.6	64.6±1.8	61.2±2.9
d7	55.7±2.4	68.0±2.3	63.5±3.2	76.0±1.6¥ * +
d28	49.0±2.5¥	71.7±2.6*	72.4±1.5*	76.4±1.5¥ *
d56	47.4±2.4¥	56.9±4.6	65.4±3.3*	68.5±2.0* $

LVESD, left ventricular end-systolic diameter; LVEDD, left ventricular end-diastolic diameter; LVEF, ejection fraction; LVFS, fraction shortening.

¥ *p*<0.05 compared to Day 1 post-infarction in the same group, one-way repeated measures ANOVA.

* *p*<0.001 *vs.* the PBS group,

$ *p*<0.05 vs. the hydrogel group and,

+ *p*<0.05 *vs.* the MSC, one-way ANOVA.

All values represent mean ± SEM.

**Figure 3:**
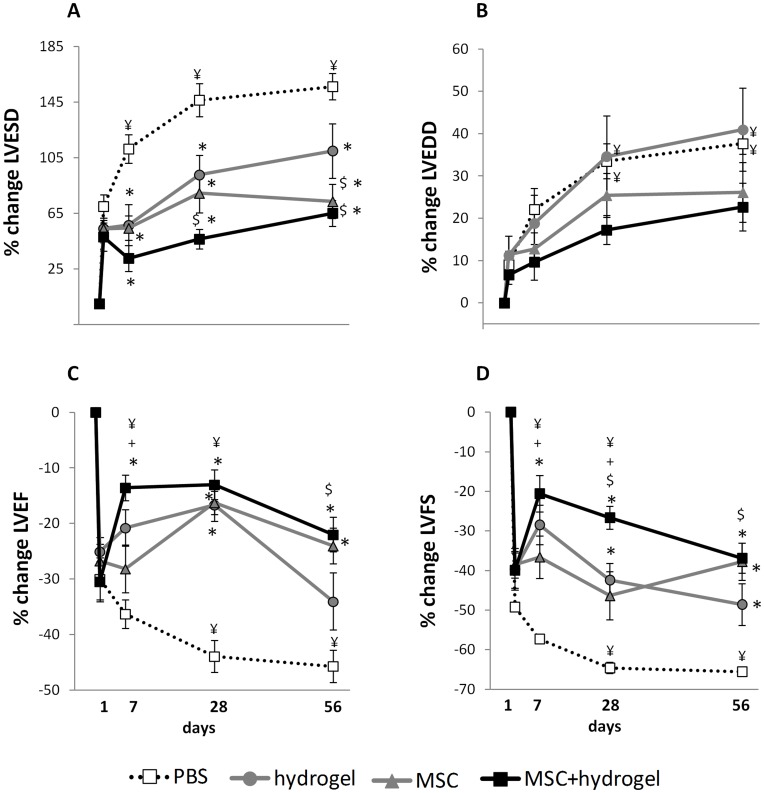
Evaluation of cardiac function by echocardiography in rats after myocardial infarction (MI). Measurements were performed at baseline before MI and 1, 7, 28 and 56 days after MI as indicated. (A) Left ventricular end-diastolic diameter (LVEDD). (B) Left ventricular end-systolic diameter (LVESD). (C) Left ventricular fractional shortening (LVFS). (D) Left ventricular ejection fraction (LVEF). ^¥^ *p*<0.05 compared to day 1 post-MI in the same group, one-way repeated measures ANOVA.*p<0.001 *vs.* the PBS group at the same time-point, *^$^p*<0.05 *vs.* the hydrogel group at the same time-point and *^+^p*<0.05 *vs.* the MSC group at the same time-point, one-way ANOVA. All values represent mean ± SEM.

As expected, MI induced an increase in LV remodeling, characterized by an increase in LV chamber dimensions, (LVESD and LVEDD) leading to a decrease in LVFS and LVEF (Figure 2, PBS conditions). As compared to the PBS group, injections of hydrogel, MSC or MSC+hydrogel, significantly attenuated the MI-induced increase in LV end-systolic diameter (LVESD) over the time period analyzed (Figure 3A). These injections also preserved the LV end-diastolic diameter (LVEDD) as compared to the PBS group, but did not improve the LVEDD (Figure 3B). The injection of hydrogel alone induced a transient but a significant increase in LVEF at Day 28, but not at Day 7 and Day 56 as compared to the PBS group. In contrast, MSC injection induced a significant increase in LVEF at Days 28 and 56 but not at Day 7, suggesting a delayed effect (Figure 3C). In addition, the LVEF did not significantly differ between the two groups throughout the study follow-up, as compared to Day 1. The injection of MSC+hydrogel during the acute phase of MI induced a significant increase in LVEF from Day 7 at Day 56 after injection, as compared to the PBS group, and a significant increase in LVEF as compared to Day 1 post MI. Interestingly, the LVEF in the MSC+hydrogel group was higher as compared to that in the MSC group at Day 7 and as compared to that in the hydrogel group at Day 56.

### Comparative Effects of Hydrogel, MSC or MSC+hydrogel on Infarct Expansion and Ventricular Fibrosis

**Figure 4:**
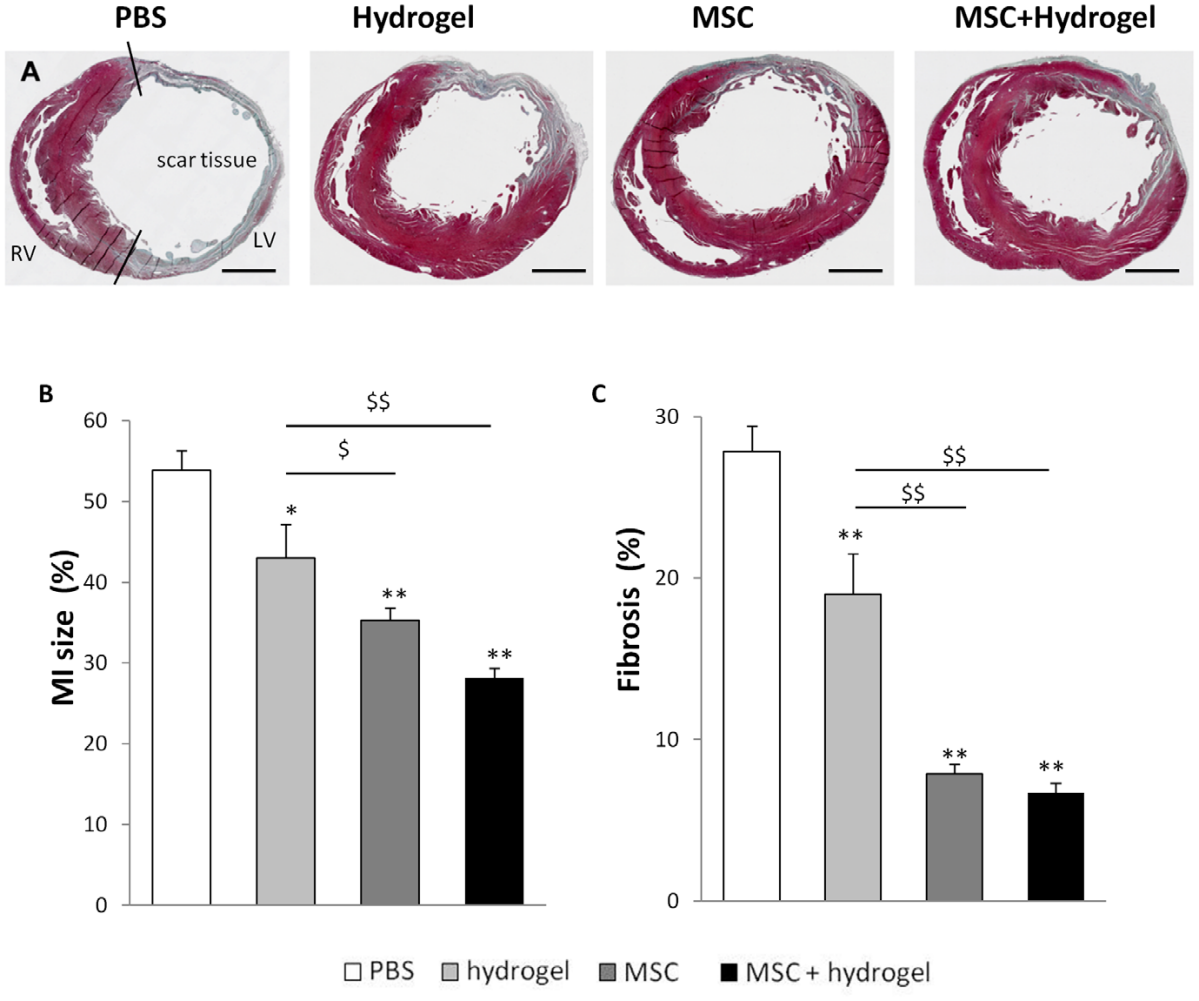
Evaluation of myocardial infarction size and left ventricular fibrosis. (A) Representative transversal histology sections of heart and Masson trichrome staining for infarct size measurement at day 56 after MI. Collagen-rich areas (scar tissue) are colored in blue and healthy myocardium in red. Scale bar = 1.5 mm. (B) Percentage of circumferential infarct size (MI size) divided by total LV tissue, and (C) percentage of fibrosis in total LV tissue. For (B) and (C): *p<0.05 and **p<0.001 *vs.* the PBS group, ^$^ *p*<0.05 and ^$$^ *p*<0.001 *vs.* the hydrogel group, one-way ANOVA. *LV, left ventricle; RV, right ventricle.* All values represent mean ± SEM.

**Figure 5:**
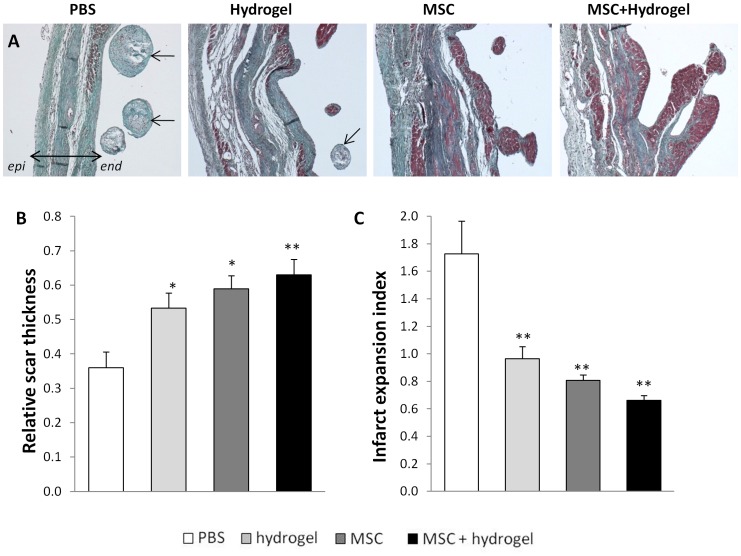
Evaluation of scar thickness and infarct expansion. (A) Representative photomicrographs of Masson trichrome staining of the scar area (collagen-rich areas in blue and healthy myocardium in red). The double arrow depicts the LV wall (*epi*, epicardium; *endo*, endocardium). The arrows show chondroid metaplasia of the endocardium. Scale bar = 0.5 mm. (B) Relative scar thickness (scar thickness/wall thickness). (C) Infarct expansion index ([LV cavity area/whole LV area]/relative scar thickness). For (B) and (C): *p<0.05 and **p<0.001, one-way ANOVA. All values represent mean ± SEM.

Morphometric analyses of heart sections were performed at Day 56 to analyze LV remodeling. For all animals, the infarct area was located in the anterior region of the left ventricle (Figure 4A). The MI size was significantly reduced in the hydrogel group, the MSC group and the MSC+hydrogel group as compared to the PBS group (Figure 4B). In addition, the size of the MI was also reduced in the MSC and MSC+hydrogel groups as compared to the hydrogel group. The percentage of ventricular fibrosis (Figure 3C) was also significantly reduced in the hydrogel group, the MSC group, and the MSC+hydrogel group as compared to the PBS group. Next, the effects of different treatments on the LV wall were assessed by measuring the relative scar thickness (Figure 5A–5B). The results showed a significant increase in the relative scar thickness in the hydrogel, MSC and MSC+hydrogel groups as compared to the PBS group. The infarct expansion index was calculated using both MI size and relative scar thickness parameters (Figure 5C). This index was significantly reduced with the injections of hydrogel, MSC and MSC+hydrogel as compared to the injection of PBS. Interestingly, chondroid metaplasia of the endocardium (indicated by arrows in Figure 4A) was often observed in the PBS group (83% of rats) whereas this feature was visible in only 14% (*p*<0.05 *vs.* PBS) of rats in the MSC+hydrogel group, 67% of rats in the hydrogel group and 60% of rats in the MSC group (*p* = NS).

## Discussion

There has been growing interest in the use of scaffolding biomaterials as a vehicle for delivery of reparative cells to improve the efficacy of targeted stem cell therapy for myocardial infarction. Among the biomaterials that have been considered for tissue engineering and regenerative medicine, hydrogels are probably the most appropriate synthetic matrices since they exhibit injectability and cross-linking properties. When considering stem cell-based cardiac tissue engineering, the ideal hydrogel should be biocompatible with respect to MSC and cardiac tissue and should also be injectable into the myocardium to provide the advantage of minimally invasive delivery [26]. In the present study, our objective was to investigate whether a self-setting cellulosic hydrogel seeded with MSC could preserve cardiac function and prevent LV remodeling during an 8-week follow-up period in a rat model of MI.

Among the various hydrogels used in cardiac tissue engineering, we focused our attention on a cellulose derivative hydrogel (Si-HPMC) that exhibits rheological properties compatible with its implantation by mini-invasive surgery [27]. The grafting of silanol groups along the HPMC chains confers this hydrogel with a self-setting property. The pH-dependent condensation between the silanol groups allows the Si-HPMC viscous solution to rapidly transform into Si-HPMC solid gel, leading to the formation of a 3D network. Interestingly, this process does not require any addition of cross-linking agents that have been extensively described as potent cytotoxic factors. The gelation time is a physicochemical parameter that has to be finely tuned, to enable successful manipulation and injection of the Si-HPMC hydrogel/cells mixture *in situ* [28]. Of interest and as previously suggested by our group, the gelation time of Si-HPMC (about 30 min) is sufficient to enable cell-hydrogel mixing [29] as well as its injection *in vivo* [30]. The need for optimizing hydrogel elasticity has also been recognized as a physicochemical parameter that governs the regenerative potential of biomaterials, supporting adequate stem cell differentiation [31] as well as the mechanical function of the targeted tissue [32]. Engler *et al.* defined a range of rigidity for various tissues measured by the elastic modulus (E). For cardiac muscle tissue, the E value ranges between 8 and 17 kPa [31]. Of note, our mechanical data showed that Si-HPMC hydrogel exhibits an E value of about 0.3 kPa, which is quite lower than that of cardiac tissue and as such, is unlikely to adversely affect the mechanical properties of the myocardium. In addition, the formation of a 3D network could provide cells with a protective environment particularly during the injection and implantation phase in cardiac tissue. Aguado et al. showed that cell protection is mainly due to the mechanical and scaffolding properties of hydrogels [33]. Indeed, crosslinked hydrogels improved cell viability during the injection in the host tissue compared to non-crosslinked hydrogels. They have also shown that extensional flow at the entrance of the syringe needle is the main cause of acute cell death. These results provide mechanistic insight into the role of mechanical forces during cell delivery and support the use of protective hydrogels in future clinical stem cell injection studies. Other key parameters in the preclinical development of innovative biomaterial-based regenerative strategies include the biological properties of biomaterials, such as cytocompatibility. Along these lines, whereas Si-HPMC has been largely described as being cytocompatible with both osteogenic [34] and chondrogenic cells [29], the data reported in the current study additionally indicate that Si-HPMC supports the 3D viability of bone marrow-derived MSC.

In light of these data, we logically embarked on *in vivo* experiments to determine the beneficial effects of injecting either hydrogel alone, MSC alone or MSC-seeded hydrogel, on the cardiac parameters of infarcted rat hearts. LV dysfunction and remodeling after MI are major determinants of transition to heart failure and cardiac mortality [4], [35]. We thus evaluated LV dysfunction by determining the LV ejection fraction (LVEF). The LV diameters, infarct size, LV fibrosis and scar thickness were then assessed to determine LV remodeling. Over the past decade, several studies have documented the cardioprotective effects of injecting hydrogels alone. For instance, the intramyocardial injection of alginate hydrogel [36] or fibrin glue [37] has been proposed as an effective acellular strategy to prevent adverse cardiac remodeling and dysfunction after MI in rats. Interestingly, in our study, the injection of Si-HPMC hydrogel alone affected the LV function mainly during the first 4 weeks after MI, with a progressive decrease thereafter. Taken together, these data suggest that the injection of hydrogel primarily preserves short-term cardiac function but is probably insufficient to prevent long-term heart failure. Similar results were observed in other studies using non-degradable hydrogels. Dobner *et al.,* injected a non-degradable PEG into the infarct immediately after MI and showed that there was temporary retardation of LV remodeling at early time points, but not at later time points [38]. Rane et al. demonstrated recently that passive structural reinforcement alone was insufficient to prevent post-MI remodeling, suggesting that bioactivity and/or cell infiltration due to degradation of injectable materials are likely playing a key role in the preservation of cardiac function [39]. The mechanism underlying the transient effect of hydrogel injected *in situ* may be related to the capability of hydrogels to increase scar thickness and stabilize the early infarct, by providing scaffolding and critical physical support to the LV wall [16]. The loss of long term efficacy could possibly be due to the absence of uniformity in polymer spread allowing for regional and global function abnormalities, as well as infarct expansion in areas where the polymer was not present. Further studies are needed to fully understand the effects of polymer spread and stiffness on infarct expansion and deterioration of cardiac function [40].

Our results showed that intramyocardial injection of MSC gives an optimal therapeutic benefit starting 4 weeks after MI. In past, numerous preclinical studies reported that intramyocardial transplantation of MSC after acute myocardial infarction improved cardiac function and decreased infarct size several weeks after MSC injection [41], [42], [43]. The recent review of Elnakish et al. reported the effects of MSC therapy on mice, rats and large animal models of MI. In rat models of acute MI, the improving LVEF was mainly observed starting from 4 weeks and up to 8 weeks [44]. The delayed effect of MSC after implantation may be due to the hypoxia, inflammation and loss of ECM support in infarcted cardiac tissue that probably lead to a poor MSC engraftment [45]. Given the short-term effect of Si-HPMC hydrogel and the delayed effect of MSC, we associated our hydrogel with MSC in order to determine whether this association may be beneficial, not only in the short-term but also in the mid-term, to prevent the detrimental consequences of MI. As expected, our results showed that the co-injection of Si-HPMC hydrogel and MSC has a marked short and mid-term effect on LV function and remodeling. Of particular interest, the co-injection of Si-HPMC and MSC had a more prominent effect during the first 7 day-acute phase period as compared to the injection of hydrogel alone. Considering that stem cells, such as MSC, normally reside in “niches”, complex 3D environments regulated by physical interactions and soluble factors [46], one can assume that hydrogel may be able to simulate this microenvironment, thereby providing cells with a protective “niche”. In support of this hypothesis, several reports have indicated that hydrogels made of fibrin or polyethylene glycol may be able to increase the survival and retention of intramyocardial transplanted cells and further improve the impaired cardiac function compared to MSC alone [47], [48]. Whether our cellulose derivative Si-HPMC hydrogel can provide cells with such a favorable environment with appropriate mechanical and biological stimuli requires further investigation.

In addition to the echocardiography parameters used to assess LV function and remodeling after MI, such as LVEF and LVFS, histological analyses may also provide further insight into the tissue changes that occur. It is recognized for a long time that the presence of focal endocardial metaplasia, consisting of chondroid tissue in areas of transmural scarring, reflects the severity of MI [49], [50]. Accordingly, the intramyocardial administration of Si-HPMC hydrogel seeded with MSC was found to limit the extent of transmural MI, preserve endocardial myocytes and reduce metaplasia. These data strengthen our hypothesis that the intramyocardial injection of hydrogel and MSC may be a relevant strategy to prevent the short-term and mid-term deleterious effects of MI on ventricular remodeling.

Despite promising results, this study exhibits limitations. First, the experiments were designed to evaluate whether a hydrogel-assisted intramyocardial injection of bone marrow-derived MSC could attenuate post-MI cardiac disorders. However, this study did not allow deciphering the mechanisms explaining the beneficial effects of this treatment on cardiac function particularly at short-term after myocardial infarction. Secondly, histological experiments showed persistence of labeled MSC injected with hydrogel until 14 days, with cell morphology similar to MSC injected alone. Unfortunately we were not able to quantitatively compare MSC survival in hearts transplanted with MSC with or without hydrogel. Nevertheless, cell survival may not be the solely mechanism underlying the effect of the hydrogel injected *in situ,* as this one may rely on capability of hydrogels to increase scar thickness and stabilize the early infarct, by providing scaffolding and critical physical support to the left ventricular wall. While a previous study performed in bone tissue suggest that the Si-HPMC hydrogel underwent phagocytosis from the edge to the center of the implantation site at 8 weeks after implantation in host tissue [51], the degradation kinetics of Si-HPMC hydrogel in cardiac tissue remains to be determined. Finally, to limit the animal number in accordance with the ethical committee, we did not include a sham animal group. Nevertheless, numerous previous animal studies showed that left thoracotomy alone does not alter cardiac function and morphology on the long term [52].

In summary, we have demonstrated that intramyocardial injection of a self-setting cellulose derivative hydrogel seeded with MSC induced short-term recovery of ventricular function and mid-term prevention of remodeling in a rat model of coronary artery ligation-induced MI. In the future, it should be defined whether these beneficial effects may be related to the specific scaffolding properties of the Si-HPMC hydrogel that may provide it with the ability to support MSC injection and engraftment within the cardiac tissue. Together with a catheter-based cell delivery system [53], the use of an injectable scaffolding hydrogel offers the possibility to prevent the damaging consequences of MI.

## Materials and Methods

### Isolation, Culture and Characterization of Bone Marrow Mesenchymal Stem Cells

Bone marrow (BM) was obtained from female Lewis rats weighing 180–200 g (Janvier, France). BM from the femur cavity was flushed with α-MEM medium (Invitrogen corporation, Paisley, U.K.) containing 10% FCS (Hyclone Perbio, Thermo Fisher Scientific), 1% L-Glutamine, 1% penicillin/streptomycin (Invitrogen) and 2 ng/mL of human basic FGF2 (AbCys, Paris, France). The cell suspension was centrifuged (1,200 rpm, 7 min) and cells were plated in culture flasks (200,000 cells/cm^2^). Non adherent cells were removed after 72 h. MSC were recovered by their capacity to strongly adhere to plastic culture dishes without cell sorting. MSC were routinely cultured and characterized by flow cytometry at passage 3 using anti-rat CD90, CD29 FITC-conjugated antibodies and anti-rat CD45, CD34, CD54, CD86 PE- conjugated. Immediately before *in vivo* injection, the adherent MSC were detached with trypsin-EDTA, centrifuged for 1 min at 1,200 *g*, and resuspended in PBS-BSA (0.1%). Aliquots containing 2.10^5^ MSC were incubated with primary antibodies for 30 min at 4°C. The suspended MSC were washed and then analyzed with a LSRI fluorescence-activated cell sorter (Becton Dickinson). For each sample, 10,000 events were acquired and analyzed with CellQuest^TM^Pro software. Results were expressed as the percentage of positive cells by comparison with the isotype-matched negative control antibodies on histogram plots.

### Silanized Hydroxypropyl Methylcellulose-based Hydrogel Preparation

#### Synthesis of Si-HPMC hydrogel

Hydroxypropyl methylcellulose (HPMC E4M®) was purchased from Colorcon-Down chemical (Bougival, France). Si-HPMC was synthesized by mixing silicium 0.5% (w/v) with HPMC to form a heterogeneous medium, as previously described [21]. Si-HPMC was solubilized in 0.2 M NaOH (3%) under constant stirring for 48 h at room temperature. The solution was dialyzed against 0.09 M NaOH using 6–8 kDa dialysis tubes (SpectraPor 1, Thermo Fisher Scientific, France). The resulting viscous solution (pH 12.6) was sterilized by autoclaving and then mixed using luer-lock syringes with sterile 4-(2-hydroxyethyl)-1-piperazineethanesulfonic acid buffer (HEPES, pH 3.6; Sigma-Aldrich, St Louis, USA) at a volume ratio of 1∶1 as previously described [21]. The final product was a hydrogel (pH 7.4) containing 1.5% Si-HPMC.

#### Rheological and mechanical measurements

Cross-linking of 1 ml Si-HPMC hydrogel was induced in 12-well plates. Dynamic rheological measurements were performed with a rotational rheometer (Rheostress 300, ThermoHaake®, Germany) using a coni-cylindrical geometry with a diameter of 60 mm and a cone angle of 1°. A multi-wave procedure was performed with 3 frequencies of 1, 3.2 and 10 Hz, with an imposed stress of 1 Pa. Oscillation tests measuring storage modulus (G’) and loss modulus (G”) were performed to study the self-setting process and gel point. Gel points, given as the time taken for the liquid (G”>G’) to turn into a solid (G’>G”), were evaluated according to the derived percolation theory [28]. Shear strain measurements were performed on 9 samples with a Haake mars. Frequencies were applied at a fixed total shear stress (1Pa) and 0.21N. Oscillation tests were performed to measure G’ and G” after 3 weeks of gelation. The Si-HPMC hydrogel compressive modulus was measured using a TA HD-Plus (Stable Micro Systems). Six samples were tested after three weeks of cross-linking and the compressive modulus was calculated on the basis of strain change from 0 to 5%.

#### Cell viability in three dimensional culture

Three-dimensional MSC viability was quantitatively assessed by Live & Dead assays (Invitrogen, France) followed by confocal imaging. MSC viability was assessed by measurement of fluorescence intensity using ImageJ (NIH) software. Briefly, MSC were trypsinized and immediately mixed with Si-HPMC hydrogel at a final concentration of 10^6^ cells/mL. They were then molded into ultra-low attachment 24-well plates and incubated at 37°C for 1 h to allow for the hydrogel to crosslink. Culture medium was then added and the MSC were cultured within the hydrogel for 24 h, 48 h and 7 days. Live & Dead assays were performed according to the manufacturer instructions (Invitrogen). Briefly, culture medium was replaced by fresh culture medium supplemented with 5 mM calcein-AM and 2 mM ethidium homodimer-1. After 10 min, the dye mixture was removed and the hydrogel was thoroughly rinsed with PBS, before being analyzed with a Nikon A1R confocal laser-scanning microscope (Nikon France) equipped with an argon laser (488 nm) and a laser diode (561 nm).

Images were recorded in 512×512 pixels with an objective CFI Plan Fluor ELWD 40X objective. For each sample, 6 random positions were chosen within the hydrogel, and a stack of 100 planes were taken from these 6 positions along the z axis using a 10 µm step size. Images obtained per sample were analyzed using ImageJ (NIH) software with the “3D object counter” plug-in. Each condition was tested in triplicate, and each experiment was repeated three times.

#### *In vivo* cell tracking

To track MSC after intramyocardial injection with Si-HPMC hydrogel, 3×10^6^ MSC were labeled *in vitro* immediately before injection using a green intracellular fluorescent dye, CFSE (carboxy-fluorescein diacetate succinimidyl ester, Molecular Probes, concentration 5 mM) as previously described [54] or the PKH26 Fluorescent Cell Linker Kits for General Cell Membrane Labeling (Sigma-Aldrich, PKH26GL, concentration 4×10^−6^ M). Cell labeling was verified by fluorescence microscopy before injection. 24 hours after *in vivo* injection of a 150 µl solution containing MSC and Si-HPMC hydrogel, rats were sacrificed and heart harvested for histological analysis. Paraffin embedded sections of 5 µm were counterstained with To-Pro-3 (Invitrogen, T3605) or Vectashield Mounting Medium with DAPI (Vector Laboratories, H-1200). For MSC specific detection, sections were successively incubated (90 min, room temperature) with biotin rat monoclonal anti-CD90 antibody diluted 1∶100 (CEDARLANE, CL005B) and HRP-streptavidin with 2-Solution DAB kit (Invitrogen).

#### Induction of myocardial infarction in rats and implantation

Animal studies were performed the agreement of the regional Animal Ethics Committee (CREEA, “Comité régional d’éthique en matière d’expérimentation animale”). Myocardial infarction was induced by coronary artery ligation and intramyocardial injections were performed as previously described [54]. In brief, Female Lewis congenic rats (180–190 g, Janvier) were anesthetized with a mix of isoflurane/oxygen inhalation (3%/97%), intubated and ventilated (Harvard Rodent Ventilator, Harvard Apparatus). A left lateral thoracotomy in the fourth intercostal space was performed to expose the anterior surface of the heart. The proximal left anterior descending (LAD) coronary artery was ligated with a 6.0 polypropylene snare (Ethicon). The area displaying tissue blanching and wall motion akinesis was identified as the infarct. Immediately after coronary artery ligation, 150 µl of a solution containing Si-HPMC hydrogel alone (hydrogel), MSC alone (3×10^6^ cells) in PBS, a combination of MSC and Si-HPMC hydrogel (MSC+hydrogel), or PBS (used as the control) were injected into the myocardium using a 26-gauge needle. A final volume of 150 µl was delivered to 3 injection sites surrounding the infarcted area.

#### Echocardiography measurements

Echocardiography measurements were performed 1 day before MI induction (baseline), and 1, 7, 28, and 56 days after MI, in anesthetized rats (2% isoflurane inhalation) using a General Electric Vivid 7VR (GE Medical System; Milwaukee, WI, USA) equipped with a 13-MHz transducer. Left ventricular end-diastolic diameter (LVEDD), LV end-systolic diameter (LVESD), and LV fractional shortening (LVFS) were recorded from the parasternal long-axis M-mode images using averaged measurements from 3 to 5 consecutive cardiac cycles in accordance with the American Society of Echocardiography guidelines [55]. Left ventricular end-diastolic and end-systolic volumes (LVEDV and LVESV) were calculated from bidimensional long-axis parasternal views taken through the infarcted area by means of the single-plane area-length method [56]. The LV ejection fraction (LVEF) was calculated as follows: LVEF = (LVEDV-LVESV)/LVEDV)×100.

#### Histopathology

Rat hearts were recovered, washed in PBS and fixed in 10% formalin. Hearts were then embedded in paraffin and 6 µm sections were cut from the apex to the level just below the ligation site. Three evenly spaced sections were stained with Masson trichrome and observed with a Nikon TE2000-E inverted microscope.

The circumferential extent of the scar to total LV tissue [57], relative scar thickness, and infarct expansion index [58] were quantified as previously described using ImageJ software (NIH). The average epicardial and endocardial infarct ratios were calculated for each section based on the measurement of epicardial and endocardial infarct lengths and epicardial and endocardial LV circumferences. For each heart, the infarct size was calculated as the mean value for the 3 analyzed sections.

Scar and septum thickness were measured at 3 different random sites and relative scar thickness was calculated as the mean scar thickness/septum thickness. The infarct expansion index was calculated as the LV cavity area/(whole LV area/relative scar thickness). The percentage area of fibrosis in the remote left ventricle was quantified using in-house image analysis software based on the equation: %fibrosis = fibrotic area/(fibrotic area+non-fibrotic area) [57].

#### Statistical Analysis

All values are shown as mean±SEM. Comparison of means was performed using one-way ANOVA followed by a post-hoc test when appropriate (Fisher’s projected least significant difference). Frequency comparisons were performed using the Fisher exact test. Echocardiography parameters measured during the 8-week follow-up period were compared between groups using one-way ANOVA and within groups using one-way repeated measures ANOVA. Comparisons were followed by post hoc tests when appropriate. *p*<0.05 was selected as the threshold for statistical significance. All tests were carried out using SigmaStat 3.5 software for Windows.

## References

[1] Cohn JN, Ferrari R, Sharpe N (2000) Cardiac remodeling–concepts and clinical implications: a consensus paper from an international forum on cardiac remodeling. Behalf of an International Forum on Cardiac Remodeling. J Am Coll Cardiol 35: 569–582. 10716457 10.1016/s0735-1097(99)00630-0

[2] Jugdutt BI (2003) Ventricular remodeling after infarction and the extracellular collagen matrix: when is enough enough? Circulation 108: 1395–1403. 12975244 10.1161/01.CIR.0000085658.98621.49

[3] Bolognese L, Neskovic AN, Parodi G, Cerisano G, Buonamici P, et al (2002) Left ventricular remodeling after primary coronary angioplasty: patterns of left ventricular dilation and long-term prognostic implications. Circulation 106: 2351–2357. 12403666 10.1161/01.cir.0000036014.90197.fa

[4] Savoye C, Equine O, Tricot O, Nugue O, Segrestin B, et al (2006) Left ventricular remodeling after anterior wall acute myocardial infarction in modern clinical practice (from the REmodelage VEntriculaire [REVE] study group). Am J Cardiol 98: 1144–1149. 17056315 10.1016/j.amjcard.2006.06.011

[5] Verma A, Meris A, Skali H, Ghali JK, Arnold JM, et al (2008) Prognostic implications of left ventricular mass and geometry following myocardial infarction: the VALIANT (VALsartan In Acute myocardial iNfarcTion) Echocardiographic Study. JACC Cardiovasc Imaging 1: 582–591. 19356485 10.1016/j.jcmg.2008.05.012

[6] Segers VF, Lee RT (2008) Stem-cell therapy for cardiac disease. Nature 451: 937–942. 18288183 10.1038/nature06800

[7] Aggarwal S, Pittenger MF (2005) Human mesenchymal stem cells modulate allogeneic immune cell responses. Blood 105: 1815–1822. 15494428 10.1182/blood-2004-04-1559

[8] Tse WT, Pendleton JD, Beyer WM, Egalka MC, Guinan EC (2003) Suppression of allogeneic T-cell proliferation by human marrow stromal cells: implications in transplantation. Transplantation 75: 389–397. 12589164 10.1097/01.TP.0000045055.63901.A9

[9] Nagaya N, Kangawa K, Itoh T, Iwase T, Murakami S, et al (2005) Transplantation of mesenchymal stem cells improves cardiac function in a rat model of dilated cardiomyopathy. Circulation 112: 1128–1135. 16103243 10.1161/CIRCULATIONAHA.104.500447

[10] Li L, Zhang Y, Li Y, Yu B, Xu Y, et al (2008) Mesenchymal stem cell transplantation attenuates cardiac fibrosis associated with isoproterenol-induced global heart failure. Transpl Int 21: 1181–1189. 18783386 10.1111/j.1432-2277.2008.00742.x

[11] Maurel A, Azarnoush K, Sabbah L, Vignier N, Le Lorc’h M, et al (2005) Can cold or heat shock improve skeletal myoblast engraftment in infarcted myocardium? Transplantation 80: 660–665. 16177642 10.1097/01.tp.0000172178.35488.31

[12] Toma C, Pittenger MF, Cahill KS, Byrne BJ, Kessler PD (2002) Human mesenchymal stem cells differentiate to a cardiomyocyte phenotype in the adult murine heart. Circulation 105: 93–98. 11772882 10.1161/hc0102.101442

[13] Niagara MI, Haider H, Jiang S, Ashraf M (2007) Pharmacologically preconditioned skeletal myoblasts are resistant to oxidative stress and promote angiomyogenesis via release of paracrine factors in the infarcted heart. Circ Res 100: 545–555. 17234963 10.1161/01.RES.0000258460.41160.ef

[14] Tang YL, Tang Y, Zhang YC, Qian K, Shen L, et al (2005) Improved graft mesenchymal stem cell survival in ischemic heart with a hypoxia-regulated heme oxygenase-1 vector. J Am Coll Cardiol 46: 1339–1350. 16198853 10.1016/j.jacc.2005.05.079

[15] Christman KL, Lee RJ (2006) Biomaterials for the treatment of myocardial infarction. J Am Coll Cardiol 48: 907–913. 16949479 10.1016/j.jacc.2006.06.005

[16] Vunjak-Novakovic G, Tandon N, Godier A, Maidhof R, Marsano A, et al (2010) Challenges in cardiac tissue engineering. Tissue Eng Part B Rev 16: 169–187. 19698068 10.1089/ten.teb.2009.0352 PMC2946883

[17] Ye Z, Zhou Y, Cai H, Tan W (2011) Myocardial regeneration: Roles of stem cells and hydrogels. Adv Drug Deliv Rev 63: 688–697. 21371512 10.1016/j.addr.2011.02.007

[18] Christman KL, Vardanian AJ, Fang Q, Sievers RE, Fok HH, et al (2004) Injectable fibrin scaffold improves cell transplant survival, reduces infarct expansion, and induces neovasculature formation in ischemic myocardium. J Am Coll Cardiol 44: 654–660. 15358036 10.1016/j.jacc.2004.04.040

[19] Leor J, Tuvia S, Guetta V, Manczur F, Castel D, et al (2009) Intracoronary injection of in situ forming alginate hydrogel reverses left ventricular remodeling after myocardial infarction in Swine. J Am Coll Cardiol 54: 1014–1023. 19729119 10.1016/j.jacc.2009.06.010

[20] Jiang XJ, Wang T, Li XY, Wu DQ, Zheng ZB, et al (2009) Injection of a novel synthetic hydrogel preserves left ventricle function after myocardial infarction. J Biomed Mater Res A 90: 472–477. 18546187 10.1002/jbm.a.32118

[21] Bourges X, Weiss P, Daculsi G, Legeay G (2002) Synthesis and general properties of silated-hydroxypropyl methylcellulose in prospect of biomedical use. Adv Colloid Interface Sci 99: 215–228. 12509115 10.1016/s0001-8686(02)00035-0

[22] Bourges X, Weiss P, Coudreuse A, Daculsi G, Legeay G (2002) General properties of silated hydroxyethylcellulose for potential biomedical applications. Biopolymers 63: 232–238. 11807750 10.1002/bip.10053

[23] Merceron C, Portron S, Masson M, Lesoeur J, Fellah BH, et al (2011) The effect of two and three dimensional cell culture on the chondrogenic potential of human adipose-derived mesenchymal stem cells after subcutaneous transplantation with an injectable hydrogel. Cell Transplant 20: 1575–1588. 21294960 10.3727/096368910X557191

[24] Mias C, Trouche E, Seguelas MH, Calcagno F, Dignat-George F, et al (2008) Ex vivo pretreatment with melatonin improves survival, proangiogenic/mitogenic activity, and efficiency of mesenchymal stem cells injected into ischemic kidney. Stem Cells 26: 1749–1757. 18467662 10.1634/stemcells.2007-1000

[25] Karaoz E, Aksoy A, Ayhan S, Sariboyaci AE, Kaymaz F, et al (2009) Characterization of mesenchymal stem cells from rat bone marrow: ultrastructural properties, differentiation potential and immunophenotypic markers. Histochem Cell Biol 132: 533–546. 19688349 10.1007/s00418-009-0629-6

[26] Singelyn JM, Christman KL (2010) Injectable materials for the treatment of myocardial infarction and heart failure: the promise of decellularized matrices. J Cardiovasc Transl Res 3: 478–486. 20632221 10.1007/s12265-010-9202-x PMC2933811

[27] Fatimi A, Tassin JF, Quillard S, Axelos MA, Weiss P (2008) The rheological properties of silated hydroxypropylmethylcellulose tissue engineering matrices. Biomaterials 29: 533–543. 17996292 10.1016/j.biomaterials.2007.10.032

[28] Fatimi A, Tassin JF, Turczyn R, Axelos MA, Weiss P (2009) Gelation studies of a cellulose-based biohydrogel: the influence of pH, temperature and sterilization. Acta Biomater 5: 3423–3432. 19481183 10.1016/j.actbio.2009.05.030

[29] Vinatier C, Magne D, Weiss P, Trojani C, Rochet N, et al (2005) A silanized hydroxypropyl methylcellulose hydrogel for the three-dimensional culture of chondrocytes. Biomaterials 26: 6643–6651. 15950277 10.1016/j.biomaterials.2005.04.057

[30] Vinatier C, Magne D, Moreau A, Gauthier O, Malard O, et al (2007) Engineering cartilage with human nasal chondrocytes and a silanized hydroxypropyl methylcellulose hydrogel. J Biomed Mater Res A 80: 66–74. 16958048 10.1002/jbm.a.30867

[31] Engler AJ, Sen S, Sweeney HL, Discher DE (2006) Matrix elasticity directs stem cell lineage specification. Cell 126: 677–689. 16923388 10.1016/j.cell.2006.06.044

[32] Engler AJ, Carag-Krieger C, Johnson CP, Raab M, Tang HY, et al (2008) Embryonic cardiomyocytes beat best on a matrix with heart-like elasticity: scar-like rigidity inhibits beating. J Cell Sci 121: 3794–3802. 18957515 10.1242/jcs.029678 PMC2740334

[33] Aguado B, Mulyasasmita W, Su J, Lampe KJ, Heilshorn S (2011) Improving viability of stem cells during syringe needle flow through the design of hydrogel cell carriers. Tissue Eng Part A 18: 806–815. 22011213 10.1089/ten.tea.2011.0391 PMC3313609

[34] Trojani C, Weiss P, Michiels JF, Vinatier C, Guicheux J, et al (2005) Three-dimensional culture and differentiation of human osteogenic cells in an injectable hydroxypropylmethylcellulose hydrogel. Biomaterials 26: 5509–5517. 15860207 10.1016/j.biomaterials.2005.02.001

[35] Poulsen SH (2001) Clinical aspects of left ventricular diastolic function assessed by Doppler echocardiography following acute myocardial infarction. Dan Med Bull 48: 199–210. 11767125

[36] Landa N, Miller L, Feinberg MS, Holbova R, Shachar M, et al (2008) Effect of injectable alginate implant on cardiac remodeling and function after recent and old infarcts in rat. Circulation 117: 1388–1396. 18316487 10.1161/CIRCULATIONAHA.107.727420

[37] Christman KL, Fok HH, Sievers RE, Fang Q, Lee RJ (2004) Fibrin glue alone and skeletal myoblasts in a fibrin scaffold preserve cardiac function after myocardial infarction. Tissue Eng 10: 403–409. 15165457 10.1089/107632704323061762

[38] Dobner S, Bezuidenhout D, Govender P, Zilla P, Davies N (2009) A synthetic non-degradable polyethylene glycol hydrogel retards adverse post-infarct left ventricular remodeling. J Card Fail 15: 629–636. 19700140 10.1016/j.cardfail.2009.03.003

[39] Rane AA, Chuang JS, Shah A, Hu DP, Dalton ND, et al (2011) Increased infarct wall thickness by a bio-inert material is insufficient to prevent negative left ventricular remodeling after myocardial infarction. PLoS ONE 6: e21571. 21731777 10.1371/journal.pone.0021571 PMC3121880

[40] Rane AA, Christman KL (2011) Biomaterials for the treatment of myocardial infarction: a 5-year update. J Am Coll Cardiol 58: 2615–2629. 22152947 10.1016/j.jacc.2011.11.001

[41] Berry MF, Engler AJ, Woo YJ, Pirolli TJ, Bish LT, et al (2006) Mesenchymal stem cell injection after myocardial infarction improves myocardial compliance. Am J Physiol Heart Circ Physiol 290: H2196–2203. 16473959 10.1152/ajpheart.01017.2005

[42] Dai W, Hale SL, Martin BJ, Kuang JQ, Dow JS, et al (2005) Allogeneic mesenchymal stem cell transplantation in postinfarcted rat myocardium: short- and long-term effects. Circulation 112: 214–223. 15998673 10.1161/CIRCULATIONAHA.104.527937

[43] Imanishi Y, Saito A, Komoda H, Kitagawa-Sakakida S, Miyagawa S, et al (2008) Allogenic mesenchymal stem cell transplantation has a therapeutic effect in acute myocardial infarction in rats. J Mol Cell Cardiol 44: 662–671. 18343403 10.1016/j.yjmcc.2007.11.001

[44] Elnakish MT, Hassan F, Dakhlallah D, Marsh CB, Alhaider IA, et al (2012) Mesenchymal stem cells for cardiac regeneration: translation to bedside reality. Stem Cells Int 2012: 646038. 22754578 10.1155/2012/646038 PMC3382381

[45] Zhang M, Methot D, Poppa V, Fujio Y, Walsh K, et al (2001) Cardiomyocyte grafting for cardiac repair: graft cell death and anti-death strategies. J Mol Cell Cardiol 33: 907–921. 11343414 10.1006/jmcc.2001.1367

[46] Morrison SJ, Spradling AC (2008) Stem cells and niches: mechanisms that promote stem cell maintenance throughout life. Cell 132: 598–611. 18295578 10.1016/j.cell.2008.01.038 PMC4505728

[47] Zhang X, Wang H, Ma X, Adila A, Wang B, et al (2010) Preservation of the cardiac function in infarcted rat hearts by the transplantation of adipose-derived stem cells with injectable fibrin scaffolds. Exp Biol Med (Maywood) 235: 1505–1515. 21127347 10.1258/ebm.2010.010175

[48] Wang T, Jiang XJ, Tang QZ, Li XY, Lin T, et al (2009) Bone marrow stem cells implantation with alpha-cyclodextrin/MPEG-PCL-MPEG hydrogel improves cardiac function after myocardial infarction. Acta Biomater 5: 2939–2944. 19426843 10.1016/j.actbio.2009.04.040

[49] Boor PJ, Ferrans VJ (1985) Ultrastructural alterations in allylamine cardiovascular toxicity. Late myocardial and vascular lesions. Am J Pathol 121: 39–54. 4050976 PMC1888030

[50] Lehoczky-Mona J, McCandless EL (1964) Ischemic Induction of Chondrogenesis in Myocardium. Arch Pathol 78: 37–42. 14148747

[51] Laib S, Fellah BH, Fatimi A, Quillard S, Vinatier C, et al (2009) The in vivo degradation of a ruthenium labelled polysaccharide-based hydrogel for bone tissue engineering. Biomaterials 30: 1568–1577. 19101030 10.1016/j.biomaterials.2008.11.031

[52] Mias C, Lairez O, Trouche E, Roncalli J, Calise D, et al (2009) Mesenchymal stem cells promote matrix metalloproteinase secretion by cardiac fibroblasts and reduce cardiac ventricular fibrosis after myocardial infarction. Stem Cells 27: 2734–2743. 19591227 10.1002/stem.169

[53] Sherman W, Martens TP, Viles-Gonzalez JF, Siminiak T (2006) Catheter-based delivery of cells to the heart. Nat Clin Pract Cardiovasc Med 3 Suppl 1S57–64. 16501633 10.1038/ncpcardio0446

[54] Fernandes S, Amirault JC, Lande G, Nguyen JM, Forest V, et al (2006) Autologous myoblast transplantation after myocardial infarction increases the inducibility of ventricular arrhythmias. Cardiovasc Res 69: 348–358. 16376327 10.1016/j.cardiores.2005.10.003

[55] Lang RM, Bierig M, Devereux RB, Flachskampf FA, Foster E, et al (2005) Recommendations for chamber quantification: a report from the American Society of Echocardiography’s Guidelines and Standards Committee and the Chamber Quantification Writing Group, developed in conjunction with the European Association of Echocardiography, a branch of the European Society of Cardiology. J Am Soc Echocardiogr 18: 1440–1463. 16376782 10.1016/j.echo.2005.10.005

[56] Folland ED, Parisi AF, Moynihan PF, Jones DR, Feldman CL, et al (1979) Assessment of left ventricular ejection fraction and volumes by real-time, two-dimensional echocardiography. A comparison of cineangiographic and radionuclide techniques. Circulation 60: 760–766. 476879 10.1161/01.cir.60.4.760

[57] Kanashiro-Takeuchi RM, Tziomalos K, Takeuchi LM, Treuer AV, Lamirault G, et al (2010) Cardioprotective effects of growth hormone-releasing hormone agonist after myocardial infarction. Proc Natl Acad Sci U S A 107: 2604–2609. 20133784 10.1073/pnas.0914138107 PMC2823907

[58] Takagawa J, Zhang Y, Wong ML, Sievers RE, Kapasi NK, et al (2007) Myocardial infarct size measurement in the mouse chronic infarction model: comparison of area- and length-based approaches. J Appl Physiol 102: 2104–2111. 17347379 10.1152/japplphysiol.00033.2007 PMC2675697

